# Hydrogel soft tissue expander for gingiva-periosteal expansion: a narrative literature review

**DOI:** 10.1186/s40902-026-00515-x

**Published:** 2026-06-02

**Authors:** Chang Youn Lee, Bongju Kim, Sung-Ho Lee, Kezia Rachellea Mustakim, Mi Young Eo, Chiyun Won, Jong-Ho Lee, Soung Min Kim

**Affiliations:** 1 Department of Oral and Maxillofacial Surgery, Dental Research Institute, School of Dentistry, Seoul National University, Seoul, Republic Of Korea; 2 Innovation Research & Support Center for Dental Science, Seoul National University Dental Hospital, Seoul, Republic of Korea; 3 Department of Oral and Maxillofacial Surgery, Hasanuddin University, Makassar, Indonesia; 4 Chiyun Won Dental Clinic, Seoul, Republic of Korea; 5 Oral Oncology Clinic, National Cancer Center, Goyang-si, Republic of Korea

**Keywords:** Hydrogel, Soft tissue expander (STE), Self-expand, Self-inflatable expander

## Abstract

**Background:**

Soft tissue expanders (STEs) are used in reconstructive surgeries to stretch tissues gradually, providing space for implants or facilitating bone augmentation. These devices have evolved from traditional silicone balloon expanders to modern self-inflating osmotic systems, which offer more controlled and less invasive expansion. This review explores the advancements in STEs, particularly the use of hydrogels, and their applications in clinical settings.

**Main text:**

Recent studies indicate that tissue expansion facilitates soft tissue growth through mechanical stretch and biological creep, enhancing vascularity and promoting fibroblast proliferation. The extracellular matrix transmits mechanical forces that influence cell behavior, while excessive pressure may lead to bone resorption, soft tissue necrosis, and expander exposure. Modern STEs include hydrogel-based, self-inflating types that absorb interstitial fluids to enable gradual expansion. These devices offer advantages such as biocompatibility, minimal scarring, and simplified surgical procedures. However, complications such as temporary deformity, hypoxia, allergic reactions, and infection remain important clinical concerns.

**Conclusion:**

Hydrogels in STEs need to balance effective expansion with minimal toxicity or morbidity. A key challenge is finding a patient-compliant, economic solution for tissue expansion before bone augmentation. Proper understanding and application of STEs can lead to successful outcomes in reconstructive surgery. Despite the usefulness of STEs, a comprehensive synthesis of their underlying biological mechanisms, clinical applications, and recent research developments is currently lacking. This review aims to fill that gap by summarizing the molecular and physical mechanisms of soft tissue growth, highlighting key factors that regulate expansion, and providing an updated overview of STEs and hydrogel technologies.

## Background

A tissue expander is an inflatable device used to create space for a future permanent implant or reconstructive surgery by gradually stretching the muscle, mucosa, and skin. In reconstructive surgery, tissue expansion involves applying mechanical stress to soft tissues, to promote the growth of additional tissue and enhances blood flow through both biological and mechanical mechanisms. Before placing dental implants, oral and dental reconstruction typically requires both vertical and horizontal bone augmentation to address large alveolar bone deficits. While guided bone regeneration (GBR) and block bone grafting can be effective, the success of these grafts depends on adequate soft tissue coverage. It is recommended to use soft tissue expanders (STEs) to ensure primary closure without tension. Contemporary STEs have progressed from the complicated inflatable silicone balloons that are periodically filled with saline to simpler, self-inflating osmotic systems that utilize hydrogels. Hydrogels expand by absorbing surrounding tissue fluids and becoming hydrated.

This review aims to provide a comprehensive synthesis of current knowledge on STEs and hydrogels. It covers the molecular and physical mechanisms underlying soft tissue growth, key regulatory factors, recent advancements in soft tissue expander (STE) research, clinical trial updates, and potential complications in oral surgery. By integrating these aspects, the review seeks to guide future research and inform clinical practice.

## Main text

### Biological mechanisms of tissue expansion

#### Tissue regeneration

Tissue expansion involves both creep and biological stretch. “Creep” refers to the phenomenon where skin continues to extend when a constant force is applied [1] “Biological stretch” describes tissue lengthening in response to applied momentary force. During expansion, tissue can be stretched while maintaining its original quality. However, if the force is excessive or applied too rapidly, it can lead to dermal rupture and the formation of striae. Argenta observed that within 24 to 48 h of injection into a tissue expander, the overlying skin softened, underscoring the importance of “creep” in the expansion process [2].

Tissue expansion induces several biological changes in the skin and underlying tissues. When skin is stretched with a tissue expander, the epidermis thickens; however, it gradually returns to its normal thickness within 4–6 weeks after the tissue expander is removed. Although the pilosebaceous units are maintained, histological analysis may reveal that they are compressed. Hyperpigmentation, caused by increased melanocyte activity during expansion, also gradually normalizes once the tissue expander is removed [3]. Histologically, the most notable effect of tissue expansion is dermal thinning. In reaction to the foreign body, a thick, fibrous capsule surrounds the expander and progressively grows. Contractile myofibroblasts, found near and within a capsule, are thought to contribute to its contraction, similar to wound contraction. Over time, the degree of capsular contracture decreases due to the pressure applied by the expander. However, the risk of contraction increases if there is an infection or if the implant becomes exposed [4]. Additionally, tissue expansion promotes alteration in elastin fibers, collagen synthesis, and increased metabolic activity [5]. Fibroblast mitotic activity is highest at initial stretching, tapering off over time. After expander removal, the dermis thickens again, and the capsule gradually resolves. The telogen phase is shortened by tissue expansion of the hair-bearing scalp, likely due to increased epidermal mitosis [6].

During skin stretching, muscle tissue experiences temporary compression, leading to a shallow concave depression, prolonged pressure can cause muscle atrophy, but it is reversible after the expander is removed [7, 8]. Tissue expansion over muscles, such as the tensor fascia lata, results in muscle lengthening and vascular growth. However, adipose tissue undergoes permanent atrophy [9, 10]. Direct pressure leads to the thinning of the cranial bone as it expands, while bone density remains unchanged [11]. Periosteal reactions and bone deformation are more common in cranial bones than long bones and typically reverse after the expander is removed. Children experience more frequent periosteal inflammation and bone thinning, and downward displacement of an expander can be observed [12–14].

#### Effect on vascularity

Tissue expansion enhances skin vascularity, as confirmed by both experimental and clinical observations. The process involves the formation of a fibrous capsule surrounding the expander, which contributes to increased vascularity, similar to the pattern in delayed flaps [15]. Temporary hypoxia induced by the expander’s pressure leads to angiogenesis, resulting in higher levels of vascular endothelial growth factor (VEGF) in expanded skin compared to the surrounding tissue [16]. During tissue expansion, mechanical stretching and biological growth result in epidermal thickening, dermal thinning, new blood vessel development, and underlying bone deformation. After the expander is removed, the skin returns to microscopic normality within a few weeks. Ultimately, tissue expansion serves to biologically resurface defects as mechanical stretching of tissue is associated with enhanced vascularity and improved cellular development [14, 17]. Kaner et al. demonstrated that laser doppler flowmetry is an effective tool for assessing microcirculation when using it to evaluate changes after biomaterial implantation [18, 19]. Several studies have reported an increase in microcirculation after insertion of tissue expanders [19, 20].

#### Cellular reactions to stretch and signaling pathways

**Fig. 1:**
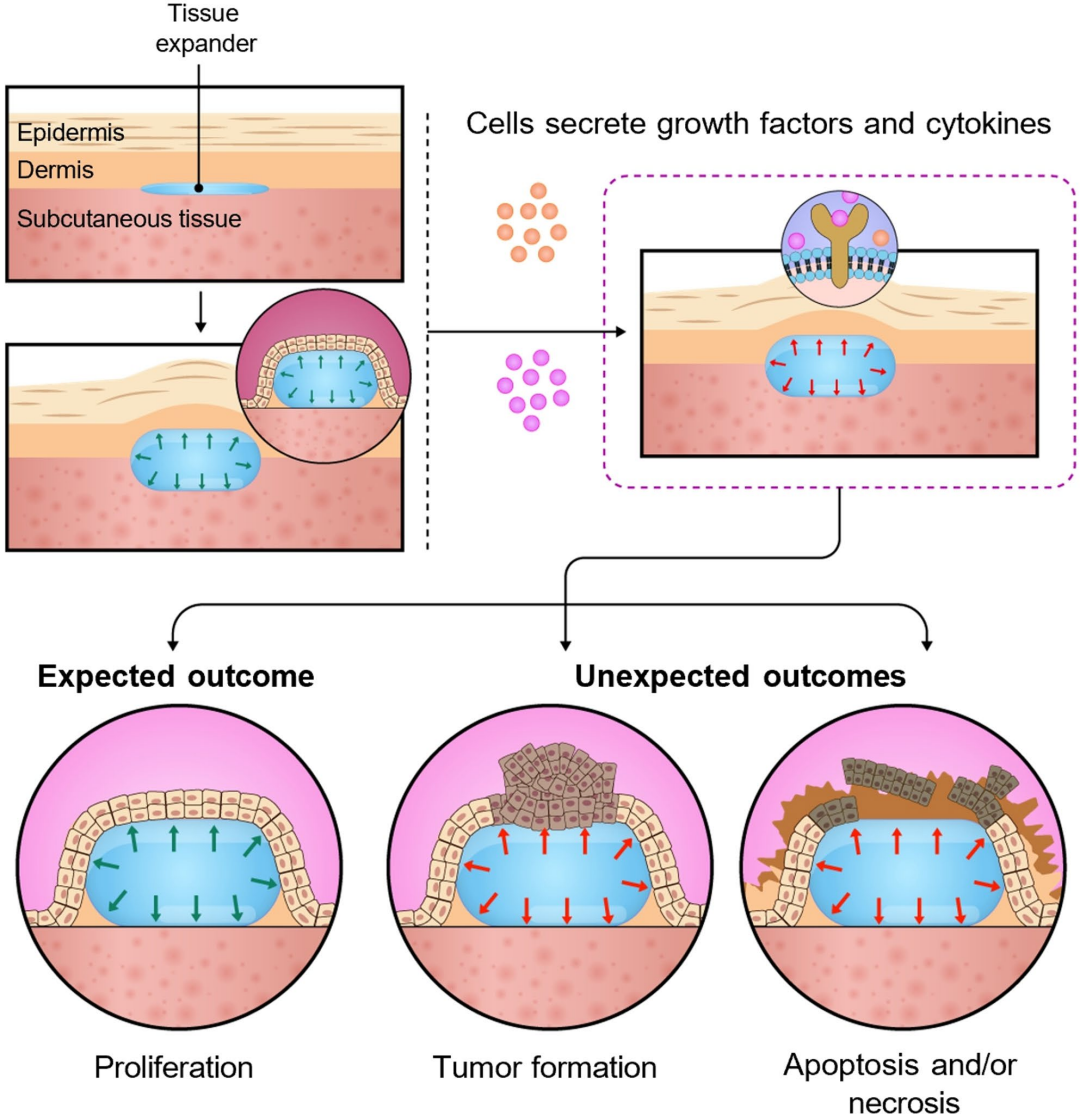
Effects of tissue expansion on surrounding tissues (Adapted and modified) [17]

Mechanical strains during tissue growth are associated with various biological processes (Fig. 1) [17]. Signal transduction is crucial in the tightly regulated tissue remodeling during expansion and any disruption in this process may lead to apoptosis, necrosis, or tumor formation. Mechanical expansion promotes cell proliferation and increases vascularity, with collagen and elastin realigning in response to strain. Various signaling mechanisms triggered by mechanical stress regulate cellular activities, including the functioning of ion channels, growth factors, and second messengers. This section discusses cellular reactions to mechanical stretching, emphasizing the skin’s viscoelastic properties and their role in tissue expansion [17]. The skin’s layered structure, consisting of the dermis, epidermis, and subcutaneous tissue, facilitates the transmission of external forces, aiding in the increase of surface area during expansion.

Research indicates that both skin elasticity and movement of interstitial fluids contribute to the increase in skin length, with cellular proliferation also playing a role. When the skin is mechanically stretched, it undergoes interstitial fluid displacement, similar to edema, and biological creep, leading to the formation of new tissue as a result of the prolonged stretching forces [21–29]. However, excessive stretching beyond the skin’s elastic limits can result in necrosis and apoptosis. Depending on the context, mechanical stretching can promote either cell proliferation or induce apoptosis, with the pathways for these responses sharing common components and influencing one another [30–33].

#### Stretch induced proliferation

Mechanical stretching triggers proliferation in cutaneous cells, particularly fibroblasts. The extracellular matrix (ECM) is crucial for this process, as it transmits external forces that deform the matrix and affect cell membranes and adhesion complexes [34–38].

Integrins play a significant role in the interaction between cytoplasmic proteins and ECM like talin, resulting in cytoskeletal reorganization. When talin attaches to integrin, it activates the receptors and enhances their binding affinity for the ECM. Activated integrins then create focal adhesion complexes connected to the actin cytoskeleton, facilitating signal transduction into the cell and initiating pathways that promote cell proliferation [39, 40]. Various growth factors present in the ECM are key regulators of this proliferation process [41, 42].

According to Jiang et al., cyclic stretching results in a large increase in fibronectin and a considerable decrease in collagen I, while static stretching increases collagen I levels while decreasing fibronectin levels [43]. This indicates that cyclic stretching inhibits human fibroblast proliferation more than does static stretching. Additionally, Qi et al. emphasized the role of nuclear envelope proteins, such as lamin A/C and emerin, which help prevent excessive proliferation of vascular smooth muscle cells induced by excessive stretching [44].

#### Apoptosis

Maintaining a balance between apoptosis and cell proliferation is essential for normal development and adaptation to environmental changes. Insufficient apoptosis can contribute to diseases like cancer, while excessive apoptosis can foster ischemic conditions and neurodegeneration. Apoptosis can be initiated by external signals from death receptors or by internal mitochondrial pathways [45–48]. In the intrinsic pathway, mechanical stretching activates the pro-apoptotic proteins Bax and Bak, leading to the release of cytochrome c and the activation of caspase 9. The binding of TNF family ligands to their corresponding receptors triggers the activation of caspase 8, initiating the extrinsic pathway [47, 49–54]. A severe type of cell death, necrosis, typically occurs in response to significant injury without caspase activation. Although apoptosis and necrosis can occur simultaneously, they exhibit distinct morphological characteristics, and the mechanism between necrosis and tissue expansion is not fully understood [55, 56].

#### Growth factors

Cell development and the restoration of the skin barrier function, and tissue integrity preservation are all regulated by the coordinated actions of multiple cell types, such as fibroblasts, keratinocytes, platelets, and macrophages in addition to a number of growth factors. Key growth factors involved in stress-induced cell growth include the FGF, EGF, TGF-β, PDGF, VEGF, CTGF, and IL families [57–63]. Research shows that IL-1, IL-6, and TNF-α levels increase during both early and chronic phases, whereas EGF, FGF-2, PDGF, VEGF and TGF-β levels increase shortly after injury but decrease during chronic conditions [64–66]. The EGF and TGF-β families of growth factors are vital for keratinocyte regulation, with EGF promoting proliferation and TGF-β inhibiting it, maintaining a balance in growth. While these factors share some downstream signaling pathways, the impact of mechanical stress on EGF and TGF-β has not been thoroughly studied [30, 63, 67, 68].

#### Ion channels

Mechanical stress on cell surfaces activates mechanosensitive ion channels and signaling molecules, with ongoing research aimed at understanding how these forces modulate these channels to produce biological signals. External forces must exert work on the channels to induce conformational changes, with stress-activated channels changing dimensions by about 4 nm between open and closed states [69–75]. These ion channels primarily include cation channels for Na⁺, K⁺, and Ca²⁺, and some anion channels like Cl⁻. The channels usually open as a result of alterations in the lipid bilayer, membrane fluidity, or tension brought on by voltage, phosphorylation, ligands, and interactions with G proteins. Mechanosensitivity varies among cell types, and elevated intracellular Ca²⁺ can trigger apoptosis. Although studies have investigated the roles of various ions in stretch responses and their connections to the cytoskeleton, the specific mechanisms involving ion channels in tissue expansion remain poorly understood [74, 76–81].

The specific role of second messenger systems in tissue expansion, particularly in epithelial cell proliferation, is not well understood [69]. However, a few late 20th-century studies emphasize how cyclic adenosine monophosphate (cAMP) regulates protein synthesis, cell development, differentiation, and proliferation, with its effects varying based on the cell type and experimental conditions [82–85]. For example, Takei et al. [85] found that cyclic strain significantly increased protein production in keratinocytes. Conversely, higher cAMP levels in skin fibroblasts were linked to decreased collagen production. Additionally, both acute and chronic cyclic strain decreased adenylate cyclase activity in cultured coronary vascular smooth muscle cells, which could lead to strain-induced cell contraction [86].

Although the precise mechanisms underlying extracellular signals remain incompletely understood, it is believed that phospholipids (PL), c-fos, and inositol phosphate (IP) transmit extracellular signals to the nucleus [30]. Molinari [87] suggested that hydrogen ions (H⁺) act as second messengers for Ca²⁺ mobilization in the IP3/Ca²⁺ signaling pathway.

### Effects of expansion pressure on STE and bone resorption

**Fig. 2:**
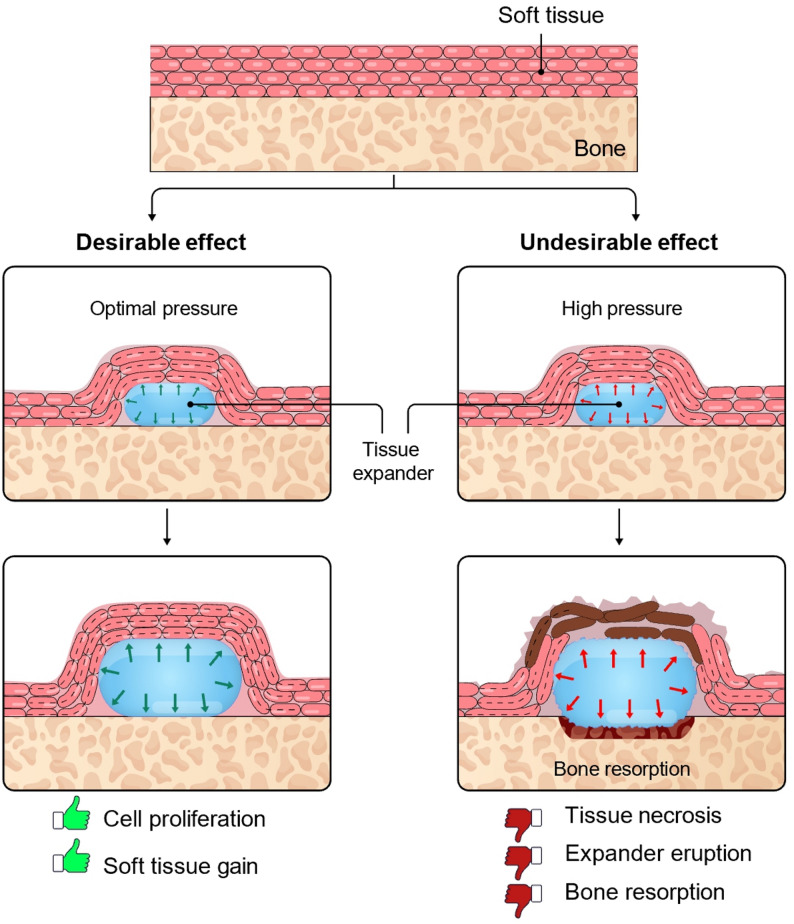
Effect of expander pressure in tissue expansion on surrounding soft tissues and underlying bone (Adapted and modified) [88]

The expansion pressure of tissue expanders can significantly affect both soft tissue expansion and bone resorption (Fig. 2) [88]. The expansion pressure associated with an expander is produced by the continuous absorption of body fluids during oral and dental reconstruction. This pressure affects both the overlying soft tissue, promoting its expansion, and the underlying bone, which may lead to bone resorption [89, 90]. Recent research [91, 92] indicates that soft tissue expansion requires a minimum pressure of 3.33 to 3.86 kPa, and a dual pressure system has been used, with a greater pressure of 13.33 kPa to promote tissue expansion after an initial pressure over the 3.86 kPa threshold [91, 92]. Through arteriolar constriction, pressures as high as 31.33 kPa can cause soft tissue expansion without necrosis, to limit the tissue’s arterial circulation [93]. While some studies suggest that STEs can promote bone gain, the general view is that they lead to pressure-dependent bone resorption [89, 90]. A preclinical study found that continuous pressure within 1.96–6.86 kPa and higher pressures caused significant resorption, whereas intermittent pressure between 9.8 and 19.6 kPa did not result in bone loss [94]. The study indicates that the continuous swelling pressure from STEs may influence bone resorption, with continuous pressure inducing it at lower thresholds than intermittent pressure. These results align with the 3.86–13.33 kPa pressure range commonly used in conventional tissue expansion [91, 92]. A recent study involving beagle dogs assessed the intraoral use of STEs and found that pressures between 7.97 and 9.61 kPa caused alveolar bone resorption [95]. These findings should be interpreted with caution, as similar pressure ranges may have different biological effects depending on the mode of application (continuous versus intermittent), duration of exposure, and tissue-specific conditions. Therefore, these values should not be directly interpreted as clinically optimal pressure ranges. In particular, while pressures above approximately 3.3–3.86 kPa are required to initiate soft tissue expansion, continuous exposure within similar or even lower ranges may still contribute to pressure-induced bone resorption. Accordingly, pressure-related outcomes in tissue expansion should be understood as context-dependent rather than defined by a single universal threshold.

### Soft tissue expanders (STEs)

**Fig. 3:**
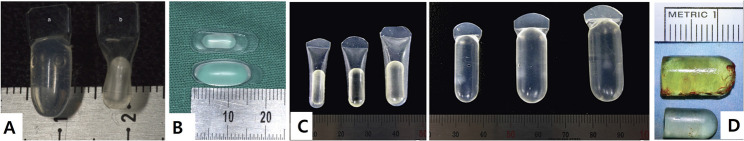
Contemporary STEs [20, 95, 96]. A Osmed^®^: left, after expansion; right, before expansion. B Tissue balloon^®^: upper, before expansion; lower, after expansion. C TissueMax^®^: three expanders of different sizes (small, medium, large) are shown in the left panel before expansion and in the right panel after expansion. D Restiex^®^: upper, after expansion; lower, before expansion [101]

STEs can be categorized into two groups [88]: (1) Those that involve consistent adjustments to sustain the required pressure for expansion and (2) Self-inflating devices designed to require no additional care once implanted [88, 96] (Fig. 3). The first category includes the simplest type of expander, typically made of a silicone balloon that is periodically filled with saline. The effects of using tissue expanders before bone grafting were documented by Lew et al. [97], Wittkampf [98], and Bahat and Handelsman [99]. According to Pietilä [100], erratic saline infusions caused hypoxia and reduced tissue perfusion due to irregular pressure. Wiese [93] reported that tissue necrosis occurred following the use of an external injection-type tissue expander on the mucosa due to the heightened risk of infection at the skin penetration site for the valve. While this method was commonly used in the past, it posed difficulties due to the need for multiple injections, resulting in considerable burden in terms of compliance for both patients and clinicians. Hydrogels can be used as an alternative to saline injection. 

#### Hydrogel as STEs

Hydrogel products are a type of polymeric material that has a significant capacity to hold water in their three-dimensional networks due to their hydrophilic structure. The widespread use of these products in various industrial and environmental applications is considered highly important. Hydrogels used in clinical applications are primarily based on copolymers of vinyl pyrrolidone (VP) and methyl methacrylate (MMA). As anticipated, synthetic hydrogels gradually replaced natural ones because of their superior water absorption capacity, longer lifespan, and the wide range of available raw chemical materials.

Materials like hydrogels, which are copolymers of VP and methacrylate derivatives, have been found to be highly biocompatible, exhibiting a typical inflammatory response followed by the formation of a thin, homogeneous fibrous capsule, which is comparable to the outcome seen with silicone balloon expanders. The thickness of this capsule is correlated with the expander pressure, and hydrogels may result in a thinner capsule because of their lower pressure [100, 102, 103].

Several studies suggest that osmotically driven devices, such as hydrogels, can effectively facilitate tissue expansion with several advantages [93]. These include avoiding tissue necrosis as hydrogels do not require hypertonic solutions, reducing expansion time using isotonic solutions, and offering greater resistance to physical stress compared to traditional balloon expanders. Hydrogels also promote tissue adaptation to their shape, which could benefit procedures like ear reconstruction. Additionally, hydrogels generate a low, consistent pressure, allowing safe, effective expansion.

Hydrogel systems create pressure by controlling osmotic pressure through the expansion of polymeric networks and mechanical contraction. Fluid absorption causes polymer elongation, which restricts swelling, while polymer interactions promote it. Together, these opposing forces help maintain expansion pressure within the system. The degree of methacrylation and crosslink density in methacrylated hydrogels is key factors influencing their mechanical properties and swelling behavior. PEGDA-based hydrogels can achieve a swelling ratio of 8–10 times that of silicone expanders and a compressive modulus between 28.3 ± 1.5 kPa and 145 ± 26.9 kPa [104]. This results from use of both low-and high-molecular weight polymers, where these of low molecular weight increase crosslinking and enhance mechanical strength while reducing expansion. Hydrogel systems with compressive moduli of 60 and 28 kPa were selected for in vivo use due to their ability to remain stable under soft tissue pressure. A high crosslinking density in HEMA/MA hydrogel expanders increases material stiffness, whereas a low crosslinking density allows greater solvent absorption and flexibility [105]. Material expansion is influenced by the base polymer’s concentration; as the concentration increases, expansion also increases. However, higher crosslinking density results in reduced expansion. The expansion capacity and mechanical strength of the STE are inversely related; hydrogels with greater expansion tend to be softer, while those with less expansion enhance mechanical properties. Importantly, the mechanical properties of hydrogels, such as compressive modulus and swelling behavior, influence not only the magnitude but also the temporal profile of pressure generation. Unlike externally inflated systems, hydrogel-based expanders typically generate a gradual and continuous osmotic pressure, which may contribute to more controlled tissue adaptation, although prolonged continuous pressure may still affect underlying bone remodeling.

Less research has been conducted on how expander size and shape influence oral and dental soft tissue expansion compared to skin repair, highlighting the need for skin studies. The choice and volume of the expander are crucial, as they influence tissue growth based on expander geometry and mechanics [106]. There is considerable variability in reported expander sizes, with some suggesting the base should match the defect size, while others recommend using expanders 2.5 to 3 times larger [107]. These recommendations are typically derived from practical experience rather than a comprehensive understanding of tissue growth.

### Contemporary STEs

Contemporary STEs have evolved from intricate inflatable silicone balloons to simpler hydrogel-based self-inflating osmotic devices, regulated by absorbing hydrates and fluids from the surrounding tissue [93]. However, it is challenging to regulate expander growth, and extrusion may perforate the surrounding soft tissue. VP/MMA copolymers are commonly used in hydrogel tissue expanders. However, these expanders have a major drawback due to their brittleness in the dry state, which limits their clinical handling. Additionally, a hydrogel may not withstand the pressure from surrounding tissues, and its reduced mechanical strength when swollen could hinder effective soft tissue expansion. A silicone envelope is required to regulate expansion and provide mechanical support, as the hydrogel’s swelling kinetics may be too rapid, even if it can withstand the pressure. A silicone-enveloped tissue expander was developed and is currently used in clinical practice [108]. Ideal expanders should be capable of producing normal tissue with minimum damage to adjacent tissues, while also fostering compliance for both patients and surgeons by reducing patient discomfort and simplifying the processes of implantation and explantation. Traditional tissue expanders using an osmotic self-inflating hydrogel are encapsulated in a silicone envelope to control the expansion rate of the hydrogel. However, this silicone envelope limits the variability in the size of the expander, and complicates control of hydrogel shape. From the initial hydrogel STE, Osmed^®^ (GmbH, Hartheim, Germany) [93], to the recently patented TissueMax^®^ (Osstem, Seoul, Korea) [95, 109] and Tissue balloon^®^ (Neobiotech Co Ltd, Seoul, Korea) [20], hydrogel-based STEs, unlike their more traditional silicone balloon equivalents, offer controlled expansion rates and manufacturer-predetermined properties, leading to improved patient compliance (Fig. 3A-C). Recent multicenter randomized clinical evidence is available for TissueMax^®^, while Osmed^®^ has also been investigated in human clinical studies [93, 95, 109]. Over the years, there has not been much significant advancement in the general design of STEs. One notable example is the creation of a reshaping self-inflating hydrogel-based system, which allows customized fit of the expander in the orodental defect area [110]. The newly developed Restiex^®^ (Akina Inc., IN, USA) has been shown to eliminate the need for a silicone membrane, maintaining a consistent expansion rate, and allowing the surgeon to cut the expander into desired sizes and shapes at the time of insertion (Table 1) [101, 111].

**Table 1:** Overview of STEs

Feature	Osmed^®^	Tissue Max^®^	Tissue Balloon^®^	Restiex^®^
Hydrogel components	VP / MMA	VP / MMA	VP / MMA	PLGA and PEG
Silicone envelope (Y/N)	Y	Y	Y	N
Mechanism of expansion	Self- inflating	Self- inflating	Self- inflating	self- inflating
Applications	Oral/maxillofacial, breast, reconstructive	Cosmetic, breast, oral/facial, bone regeneration	Oral/maxillofacial	Reconstructive, breast, oral/maxillofacial
Volume changes	4.66-6 times gain	4.67-6.25times gain	4.12 times gain	2 times gain
Initial -> final volume	0.045 ml -> 0.24 ml 0.15 ml -> 0.7 ml 0.25 ml -> 1.3 ml 0.42 ml -> 2.1 ml)	0.15 ml ->0.7 ml 0.2 ml ->1.0 ml	0.08 ml -> 0,33 ml	Not announced (size: 5 mm wide$\:\times\:$20 mm long$\:\times\:$3 mm thick)
Duration of use	6–12 months	6–8 weeks	4–8 weeks	Up to 6 months
Risk of complications	Low (infection, displacement)	Low (capsular contracture)	Low(displacement, ulcer)	Low (gradual expansion)

*MMA* Methyl methacrylate, *VP* Vinyl pyrrolidone, *PLGA* Poly lactide-co-glycolide, *PEG* Polyethylene glycol

The consistent expansion of Restiex^®^ is achieved using polylactide-co-glycolide (PLGA), a hydrophilic, biodegradable polymer traditionally used in sutures and drug delivery systems (Fig. 3D). The PLGA hydrolyzes into lactic and glycolic acids, while polyethylene glycol (PEG), a non-degradable hydrophilic polymer with minimal immune response, provides excellent biocompatibility. The amounts of PLGA and PEG are adjusted to regulate the expansion rate of the hydrogels. However, these techniques require surgery for implantation and are often associated with post-implantation complications, such as infections or mucosal rupture. Additionally, patients face significant financial strain due to the surgical procedures and associated clinical issues. Less invasive techniques are required for the implantation of expanders that can be customized to match the orodental defect shape.

### Surgical procedure of STEs

**Fig. 4:**
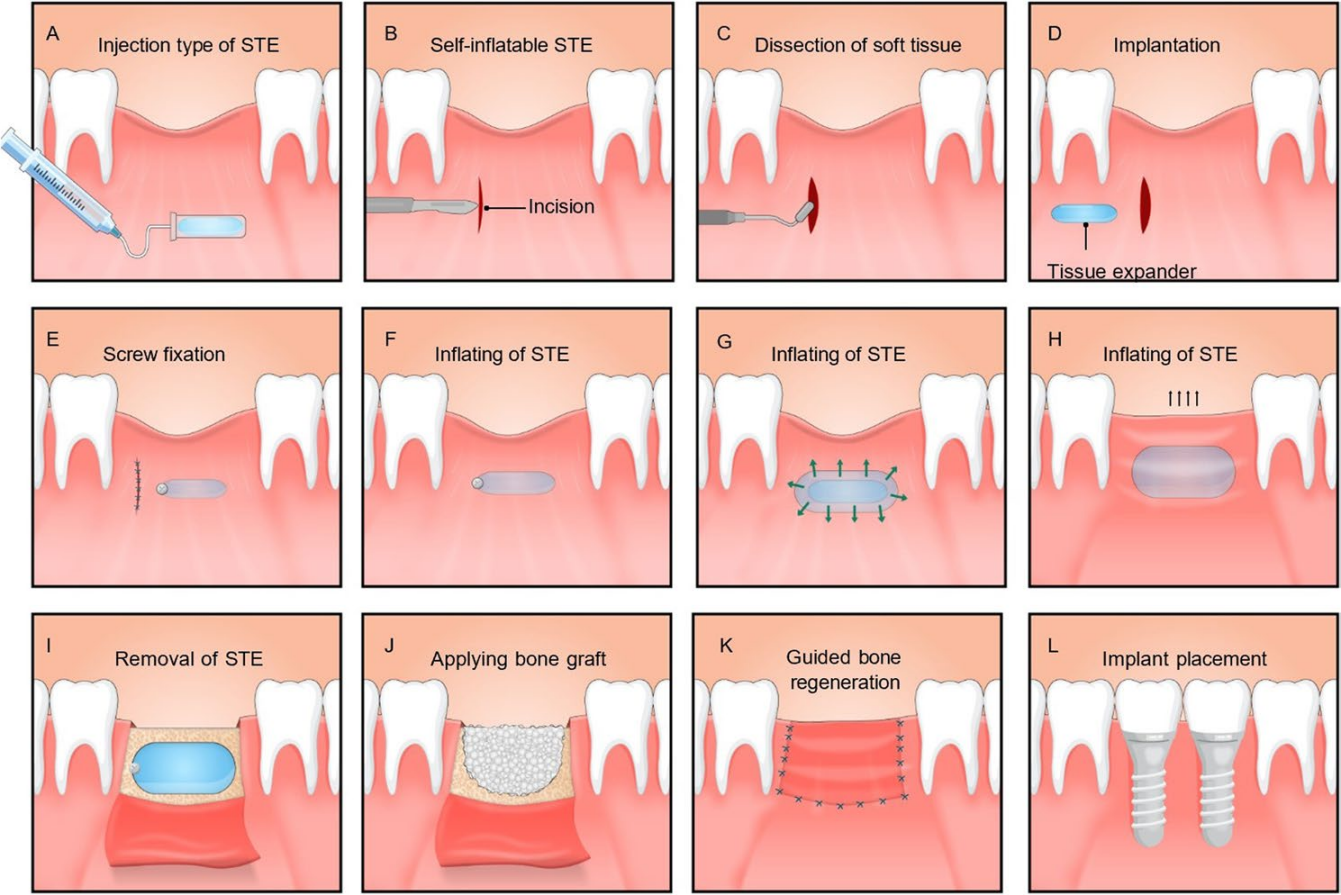
Schematic representation of the various steps involved in STE for vertically atrophied alveolar ridges (Adapted and modified) [113, 114, 118]

The surgical procedure begins with local anesthesia. A small incision is made using a scalpel and scissors or manufacturer instrument to make it easier to install and remove the tissue expander without causing any damage to the periosteum. The incision is wide enough to accommodate the expander on both sides of the defect area. Alternatively, to create a pathway on the lateral side of the defect, the incision can also be made distal to the bone level, away from the gingival margin connected to the tooth. Next, the tissue expander is implanted next to the defect location, under the gingiva or mucosa. A bone fixation screw and fine monofilament sutures are used to secure the expander in place, ensuring it remains properly positioned on the bone and within the gingiva [112]. Following the expansion period, the expander is removed by making a small incision on the lingual or palatal side of the tissue. (Fig. 4) [113, 114]. This method offers advantages over subperiosteal expander placement because it minimizes expansion of the periosteum. Instead, a network of connective tissue forms after expander’s implantation, resulting in a new underlying periosteum [90, 115–117]. Additionally, this approach does not lead to significant bone resorption, which can occur with subperiosteal implantation, and allows more controlled and less invasive bone and tissue expansion compared to traditional techniques.

The expansion phase lasts approximately 40 to 60 days and is driven by a swelling mechanism, where water enters and diffuses through the hydrogel via capillary action. The hydrogel inflates itself as a result of network relaxation and swelling as well as the interaction between water and the polymer [113].

Pain management is essential in tissue expansion, and wound care is conducted to minimize complications. Antibiotics such as amoxicillin or clindamycin are administered before and after surgery, along with ibuprofen for pain management according to patient’s age and weight. Patients are also instructed to rinse with 0.2% chlorhexidine for oral hygiene until the sutures are removed, typically two weeks post-surgery. Expansion typically continues for 6–8 weeks until the expander reaches its maximum volume, followed by bone augmentation. Before implantation, any pre-existing infections should be treated, and tissue expanders should not be used for longer than six months [112].

### Advantages and disadvantages of STEs

Compared to other similar techniques, tissue expanders offer several benefits, as below [103, 118–120].

1. Simple surgical procedure with a low incidence of complications and infections.
2. Application of biocompatible substances that replicate the texture, color and sensation of surrounding tissues.
3. Minimal risk of trauma and scarring from incisions.
4. Shorter surgical time, which helps reduce post-operative pain
5. Lower cost compared to other surgical methods
6. Remote and smaller incisions that are strategically placed away from the expansion site, minimizing incisional tension during the expansion phase and lowering the possibility of expander extrusion.

However, there are some drawbacks to be considered as below, too [113, 119, 121].

1. Temporary deformities during the expansion process, with a potential need for further treatments to address complications such as excessive bleeding or scarring.
2. Hypoxia may occur if the tissue is expanded too quickly, causing high-pressure peaks within the expander.
3. Allergic reactions can occur to the materials used in the expander.
4. Risk of infections around the expander, which may require removal of the implant
5. Inadequate tissue generation, potentially leading to ulcer, graft exposure and loss of grafted bone after surgery.

### Indications and contraindications of STEs

Tissue expansion is commonly used in several surgical procedures, as indicated below [119, 122–124].

1. Augmentation of resorbed edentulous ridges to improve the structure for dental implants.
2. Bone regeneration and onlay grafting procedures to enhance bone volume and support.
3. Soft tissue coverage and healing during cleft lip repair.
4. Treatment of decubitus ulcers using local flaps for tissue regeneration.
5. Development of post-auricular skin for external ear reconstruction.
6. Extension of forehead skin for use in forehead flap total nasal reconstruction.

But, it can be recommended to be avoided in patients with below situations [125].

1. Psychological disturbances that could affect the ability to tolerate the procedure.
2. Active infections in the body, which could increase the risk of complications.
3. Poor vascularization in the implantation area, which could hinder proper healing and expansion.
4. Systemic diseases such as hypertension, diabetes mellitus, chronic liver disease, renal failure, or bacterial pneumonia that could interfere with the body’s ability to heal and recover properly.

### Anatomical considerations for STEs use

Understanding the anatomical features of the area that needs soft tissue expansion is essential for choosing the appropriate tissue expander size, shape, and orientation. For expansion to be successful, the expander must be positioned and aligned correctly.

Studies show that tissue expanders work most efficiently when placed along Langer’s lines, which are the natural orientations of collagen fibers in the skin [126, 127]. Gingiva is more resistant to deformation than alveolar mucosa due to differences in tissue composition [128]. In dental procedures, STEs are commonly placed either subperiosteally or supraperiosteally. Submucosal placement is easier, more comfortable for patients, and better tolerated, which makes it the preferred method in many cases [95].

## Discussion

### Clinical implications of STEs

**Fig. 5:**
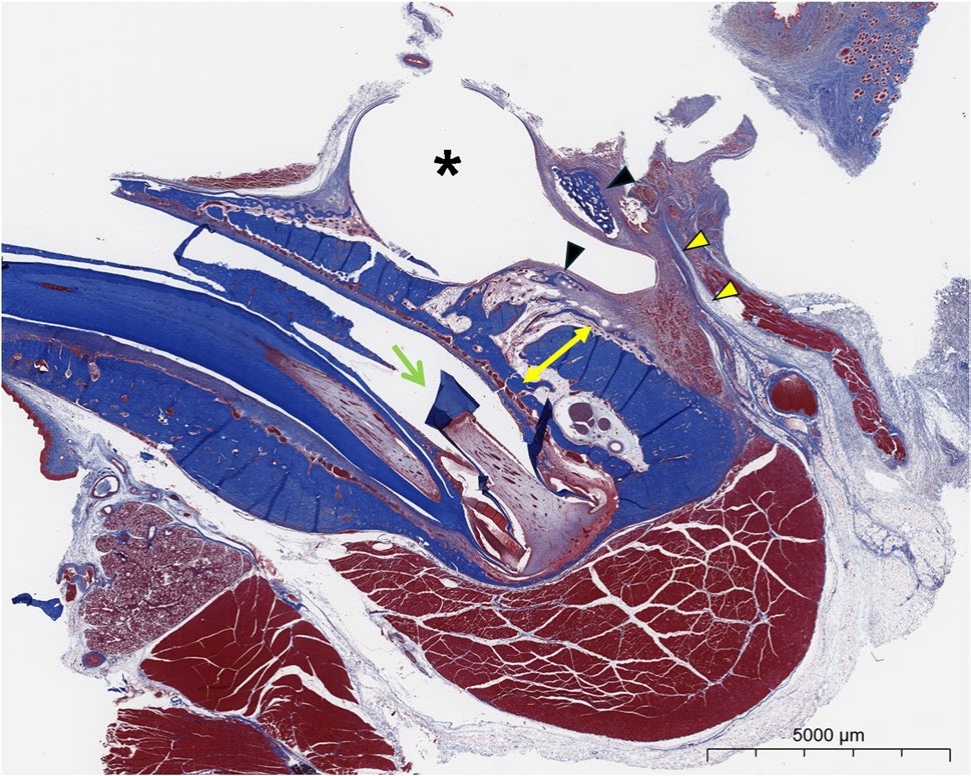
Photomicrograph of the rabbit’s active expansion area 21 days post-operative, showing expanded hydrogel and silicone (asterisk), new bone formation (black arrow head), raised periosteum (yellow arrow head), mature bone (double arrow), and a tooth germ (single arrow) on MT assay [20]

Lee et al. [20] reported an animal study using a hydrogel STE (Tissue balloon®; Neobiotech Co Ltd, Seoul, Korea) in rabbits and beagles (Table 2). A hydrogel STE was used to apply the self-inflating expander to nine adult rabbits via the submandibular approach. The hydrogel STE was inserted beneath the lateral mandibular periosteum. The expanders were removed at two, three, and four weeks, and bone and soft tissue samples were gathered for examination. The characteristics of the expander were measured and analyzed through histomorphometry. In the rabbit study using the hydrogel STE, volume, weight, and size all increased significantly over the course of the four weeks. After implantation, expansion peaked two weeks later and then gradually stabilized with swelling ratios of 107.79 ± 9.80%, at two weeks, 114.33 ± 21.06% at three weeks and 114.07 ± 34.77% at four weeks. The initial measurements were 3.36 ± 0.11 mm in width, 8.43 ± 0.43 mm in length, 3.50 ± 0.37 mm in depth, 0.13 ± 0.01 g in weight, and 0.08 ± 0.01 cc in volume. After four weeks post-implantation, these values increased to 6.07 ± 0.31 mm in width, 13.35 ± 0.58 mm in length, 5.99 ± 0.37 mm in depth, 0.34 ± 0.03 g in weight, and 0.33 ± 0.31 cc in volume. Histological examination revealed that the expanders were encased in collagen-rich tissue and exhibited no signs of inflammation (Fig. 5). A small amount of bone resorption was seen as a result of the expansion pressure. These results demonstrate the material as a biocompatible STE.

**Table 2:** Summary of in vivo studies using hydrogel osmotic expander

Author/Year	Animal model (Sample size)	Groups	Tissue expander	Duration	Placement	Clinical Outcomes	Complications
Kaner et al. (2015) [19]	Beagle dogs (*n* = 10)	Soft tissue expansion followed by GBR	Osmed^®^ hydrogel; Cylinder 0.7 mL	35 days	Submucosal (mandible)	Primary closure achieved, while there was need for releasing incisions in control group; test sites showed a significant better perfusion than control sites.	8 dehiscences in control groups
Yoo et al. (2018) [95]	Beagle dogs (*n* = 5)	Single group	Osstem 2nd-generation STE, 0.15, 0.25, 0.42 mL	28 days	Subperiosteal; screw-fixed	5.0, 5.2, 4.7 times expansion, respectively; collagen & vessels; no inflammation; alveolar resorption; collagen fibers blood vessel formation was observed	
Barwinska et al. (2017) [101]	Beagle dogs (*n* = 9)	Four implantation sites	Restiex^®^ 5 × 20 × 3 mm semicylinder	42 days	Submucosal (buccal ridge)	Slow expansion; 2 times volume change; The average distance of STEs shift was 2.7 mm. The analysis of the images showed a consistent linear expansion of 32%, which translated to 107% gain of oral mucosa linear dimension; good perfusion	3% extrusion
Garner et al. (2019) [110]	Beagle dogs (*n* = 9)	Four implantation sites, 2 expansion cycle,	Restiex Hydrogel 5 × 20 × 3 mm semicylinder	42 days	Submucosal (maxilla/ mandible)	Symmetry maintained; 45% volume gain; thin capsule; no migration; the first and second insertions resulted in linear oral mucosa gain of 8.13 mm, and 6.44 mm	First and second insertion hydrogels erupted from 4% of the first expansion sites, and 3% of the second expansion sites.
Lee et al. (2024) [20]	Beagle dogs(*n* = 5)	Soft tissue expansion only Four implantation sites	Tissue balloon^®^ Initial: 3.36 × 8.43 × 3.50 mm; 0.13 g; 0.08 cc	21 days	Subperiosteal; screw-fixed (maxilla/mandible)	Good perfusion; fibrosis, cortical bone reduction, and gingival thickening were observed; The initial measurements (3.36 × 8.43 × 3.50 mm; 0.13 g; 0.08 cc) increased to 6.07 × 13.35 × 5.99 mm, 0.34 g, and 0.33 cc.	Dehiscence in 9 sites;

In that same study, all premolars were removed from five beagle dogs. Three months following the extractions, tissue expanders were placed adjacent to the mandibular and maxillary extraction sites; they were retrieved after three weeks. Before and after inflation, gingival parameters were recorded, and a laser doppler flowmeter was used to quantify the blood perfusion in the oral mucosa at various time intervals. The dogs were euthanized after three-week expansion and tissue samples were harvested for histological examination, focusing on inflammation and bone formation. Dehiscence occurred in nine cases, with exposed sites showing granulation tissue and edema, but no pus formation. This was attributed to mechanical irritation in the expansion area rather than infection. The expansion pressure caused dehiscence and expander loss, particularly at sites opposite to the fixation, potentially exacerbated by uncontrolled chewing during the healing process. Histologically, no infection was observed, but fibrosis was observed in all cases. Assessment of the recipient bones indicated cortical bone reduction, likely due to pressure from expansion (Fig. 6). In some cases, bone formation occurred toward the medulla near the graft site. No signs of infection or dehiscence were observed in the wounds. While the exact thickness could not be measured after the expander was removed, the results showed considerable gingival thickening, even after excluding edema and granulation tissue. To evaluate changes in the area at the same position, the model was scanned both before and after surgery and images were superimposed. After three weeks post-procedure, an increase in area was observed with the initial volume of the expander increasing from 0.08 ± 0.01 cc to 0.28 ± 0.07 cc. Additionally, the surface area of the gingiva grew from 11.54 ± 3.20 cm² to 12.56 ± 3.80 cm². Baseline measurements of blood perfusion were similar across control locations, while blood flow at the test sites significantly decreased after administration of local anesthetic. However, perfusion was increased at the test sites after three days and three weeks, suggesting that the expansion process had no adverse effects on microvascular circulation [20].

**Fig. 6:**
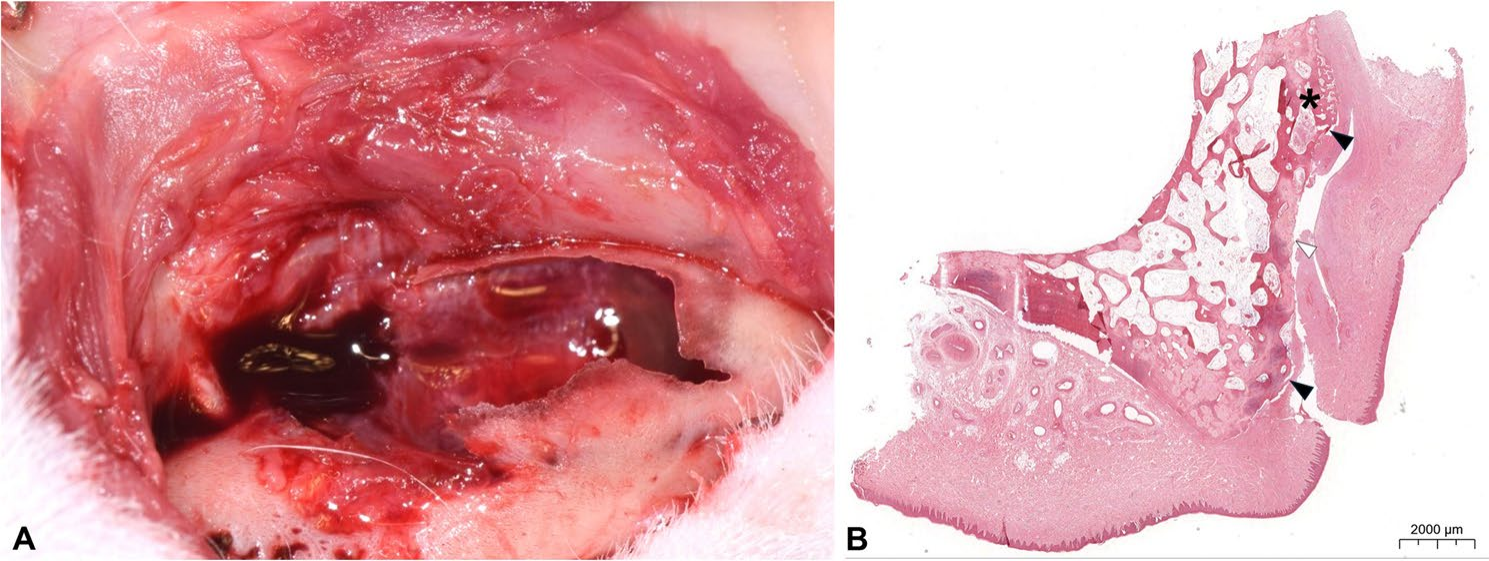
A The tissue expander was surrounded by newly formed bone after expansion for three weeks. B Expansion area of the hematoxylin and eosin-stained maxilla of a beagle (scalebar = 5 mm). The asterisk identifies new bone formation toward the medulla, the black arrowhead identifies bone formation toward the cortical bone, and the white arrow identifies cortical bone atrophy [20]

Won [129] reported a 65-year-old female who received a STE to restore a severely atrophic edentulous ridge of the mandible in 2014. The site demonstrated the importance of effective use of fixed dental implants to restore a highly atrophic mandibular edentulous patient. Without requiring an autogenous bone graft, the space generated by the tissue expander allowed effective regeneration, with a maximum vertical increase of 7.6 mm.

Despite a few animal studies, the use of Osmed^®^ tissue Expanders for soft tissue expansion prior to bone augmentation has been investigated in three human trials, including two case series and one randomized trial [112, 130, 131]. The research indicates that the tissue expanders, placed either subperiosteally or submucosally, effectively prevented bone graft exposure, tissue dehiscence, and bone resorption. However, soft tissue perforations were observed. Despite the positive outcomes, the small sample sizes of these studies limited the ability to generalize the findings. Larger clinical studies with extended follow-up are needed to gain a deeper understanding of how tissue expansion impacts vascularization, bone resorption, and risk of perforation. Additionally, the restricted shapes and sizes of the tissue expanders prevented them from being tailored to the specific needs of each patient (Table 3).

**Table 3:** Summary of clinical studies on pre-augmentation soft tissue expansion using hydrogel osmotic expander

Author/Year	Sample Size / Design	Expander Design	Placement	Duration	Clinical Outcomes	Complications(number)	Bone Grafting / Flap	Additional Findings
Kaner & Friedmann (2011) [112]	Case series *n* = 12; soft tissue expansion → bone augmentation	Osmed^®^ hydrogel; Hemisphere 0.35 mL final volume / Cylinders 0.24, 0.6, 1.3 or − 2.1 mL final volume	Submucosal	60 days	Vertical gain 7.5 ± 2.4 mm; bone volume ratio 0.1614 ± 0.0582	Perforation (2)	Vertical augmentation; tension-free closure	Encapsulation; no bone resorption
Abrahamsson et al. (2012) [131]	RCT *n* = 20 (10 experimental group / 10 control group)	Osmed^®^ hydrogel; 2.5 × 7.5 × 3 initial → 5.6 × 11 × 6 mm final	Sub-periosteal	14 days	Soft tissue gain 2.9 ± 1.1 mm; lateral/vertical bone changes recorded	Perforation (2)	Autogenous block graft; periosteal release only in control	Greater resorption vs. control at 6 months
Mertens et al. (2015) [130]	Case series *n* = 8	Osmed^®^hydrogel; 0.045–0.42 → 0.24–2.1 mL	Sub-periosteal	20, 40,90 days (based on size defect)	0.7–2.1 mL soft tissue gain	Perforation in trauma and cleft patients (2)	Autogenous/synthetic grafts	Good soft tissue, no keratinized gingiva
Byeon et al. (2020) [109]	RCT *n* = 75 (25/25/25, TET/TEG/control group)	TissueMax^®^ hydrogel; 0.15, 0.2 → 0.7, 1 mL	Sub-periosteal	28 days	TET and TEG vertical expansion 5.62 mm, horizontal expansion 6.03 mm TEG vertical gain 5.13 ± 1.32 mm	Minimal	GBR or tunneling graft	Control showed highest resorption
Byeon et al. (2020) [132]	RCT *n* = 46 (23/23)	TissueMax^®^ hydrogel; 0.08, 0.15, 0.2 → 0.5, 0.7, 1 mL	Sub-periosteal	28 days	Buccal 6.88 ± 1.64 mm; horizontal 6.65 ± 1.38 mm	Over-expansion (2); scar perforation (1)	GBR or tunneling graft	No capsule formation if removed early

*RCT* Randomized clinical trial, *TET* Tunneling graft, *TEG* Traditional bone graft, *GBR* Guided bone regeneration

TissueMax^®^ was assessed in a dog model, showing its ability to effectively generate soft tissue while maintaining mechanical stability, similar to Osmed^®^ expanders. TissueMax^®^ was used in two multicenter randomized clinical trials for soft tissue augmentation in vertically atrophied alveolar ridges [95]. The trials compared three groups: one with no tissue expansion, one with tissue expansion combined with tunneling graft (TET), and one with tissue expansion combined with traditional bone graft (TEG). TissueMax^®^ expanders were placed subperiosteally and secured with screws to prevent displacement. After four weeks, both the TET and TEG groups achieved significant tissue expansion (5.62 mm vertically, 6.03 mm horizontally) and underwent bone grafting. Six months later, implants were placed, showing no tissue thinning and minimal risk of wound dehiscence. Cone beam CT scans revealed greater vertical bone gain and lesser bone resorption in the TET and TEG groups compared to the control group. The TEG group showed a significant vertical bone gain of 5.15 ± 1.32 mm compared with the control group [109]. The study suggests that combining soft tissue expansion with conventional GBR improves bone augmentation outcomes.

The second study investigated the use of a self-inflating TissueMax^®^ expander for vertical bone augmentation [132]. After the expander was screwed into place, a tunneling bone transplant surgery was performed. All patient groups underwent implant placement after a six-month period. The expander was placed buccally rather than at the crestal region because of the rough edges of the atrophied ridges. This led to a 6.9 mm vertical soft tissue expansion and a 6.65 mm horizontal expansion. Vertical bone resorption was significantly reduced in the expander-treated patients (1.57 mm) compared to the control group (2.32 mm). However, challenges arose with the tunneling bone graft procedure, including low graft stability and difficulty assessing graft adequacy; in addition, implant success and cosmetic outcomes were not assessed. The authors recommended further randomized controlled trials with larger sample sizes and longer follow-up to assess all clinical outcomes.

### Key findings and synthesis

In the present study and previous investigations, hydrogel-based tissue expanders exert a continuous swelling pressure that effectively promotes soft tissue growth. Continuous swelling pressures in the range of 1.96–6.86 kPa have been reported in preclinical studies; however, these values should not be interpreted as clinically recommended or inherently safe ranges. While pressures above approximately 3.3–3.8 kPa are necessary to initiate soft tissue expansion, continuous exposure within similar or even lower ranges may still induce bone resorption depending on duration, biological conditions, and placement technique. Therefore, these pressure ranges should be regarded as experimentally observed values rather than definitive clinical targets, and their interpretation requires careful consideration in clinical practice.

In this context, slow and controlled expansion, rather than achieving a specific pressure threshold, appears to be more critical for balancing soft tissue augmentation and preservation of underlying bone. For example, osmotic expanders such as Osmed^®^ systems have been associated with relatively low and gradual pressure generation, and strategies such as the use of pressure-distributing structures (e.g., titanium mesh) have been suggested to mitigate localized stress on the underlying bone. However, clinical application may vary depending on anatomical and surgical conditions. Soft tissue expansion and bone response are governed by a complex interaction of pressure magnitude, duration, application pattern, and tissue-specific biological responses, rather than by a single pressure threshold. In terms of surgical placement, submucosal insertion maximizes soft tissue expansion, whereas subperiosteal insertion may be preferred when simultaneous soft tissue expansion and bone formation are desired. These findings highlight the importance of carefully controlling the swelling pressure and selecting the appropriate insertion plane to balance soft tissue augmentation with preservation or enhancement of underlying bone.

When considering tissue expander products, three types are often referenced: Osmed^®^, TissueMax^®^, and Tissue balloon^®^. The selection of type and size should be based on the size of the expansion site and the desired final volume. Among these, TissueMax^®^ and Tissue balloon^®^ exhibit faster expansion rates and are suitable for applications requiring expansion within 8 weeks. In contrast, Osmed^®^ expands more slowly and is more appropriate for gradual expansion over a period of up to 6 months. Their current availability for intraoral and dental indications varies by product, market, and regulatory status.

### Complications

The primary complications associated with hydrogel-based tissue expanders include discomfort during expansion, soft tissue perforation, and hydrogel loss. Discomfort is generally mild to moderate and related to the continuous swelling pressure exerted on the surrounding soft tissue.

Several clinical studies have reported cases of soft tissue perforation associated with hydrogel-based tissue expanders. In a study by Kaner and Friedmann, 2 out of 12 patients experienced perforation, which was attributed to infection or an oversized expander [112]. Abrahamsson et al. reported 2 cases of soft tissue perforation among 20 patients, occurring at the projection of the expander through the incision line [131]. Mertens et al. study observed 2 cases among 8 patients, both of whom had a history of trauma or previous cleft surgery [130]. More recently, Byun et al. study reported one case of mucosal perforation among 23 patients, which was related to a pre-existing scar. These findings highlight the importance of careful patient selection, proper expander sizing, and attention to previous surgical or traumatic history to minimize the risk of soft tissue perforation [132]. The Restiex^®^ in the current study causes slow, steady expansion of oral mucosa with only a 3% incidence of device extrusion [101].

If soft tissue perforation occurs, the expander should be immediately removed, and appropriate inflammation management should be provided. After allowing the tissue to fully recover, re-insertion of the expander can be attempted. Proper healing and inflammation control help prevent further complications and ensure safer subsequent tissue expansion.

### Limitations

Several limitations of this review should be acknowledged. First, the included studies often involved small sample sizes, which may limit the generalizability of the findings. Second, there was considerable heterogeneity among studies in terms of study design, outcomes, and protocols. Finally, as a narrative review, this study does not provide a quantitative synthesis, which may limit the ability to draw firm conclusions. Future research should aim to address these limitations by including larger, more homogeneous study populations and applying standardized methodologies to provide more robust evidence.

### Future directions

Modern osmotic tissue expanders primarily utilize synthetic VP/MMA polymers, but these have limitations, including immune reactions and fibrous encapsulation. Naturally derived biopolymers could offer a better alternative by avoiding immune responses and promoting tissue regeneration. Most modern STEs require invasive surgery, increasing risk of infection and patient discomfort. Injectable hydrogels, especially hyaluronic acid (HA), are a promising minimally invasive alternative that can effectively cover irregular defects and efficiently deliver growth factors [133–135]. HA has shown success in oral tissue regeneration and other applications, such as skin expansion prior to ear reconstruction, and could be adapted for orodental surgeries by modifying hydrogels to exhibit in situ swelling for more controlled tissue expansion. The integration of HA-based bioinks with three-dimensional (3D) bioprinting technology offers a promising strategy for the fabrication of tissue expanders that surpass conventional synthetic devices in terms of biocompatibility, biodegradability, and regenerative potential [136]. HA, a naturally occurring polysaccharide in the extracellular matrix, provides excellent cytocompatibility and can be chemically modified to tailor its mechanical properties, gelation kinetics, and degradation rates, which are critical for designing tissue expanders capable of sustained, controlled expansion. These constructs can be printed with controlled porosity and embedded with stem cells or growth factors to promote vascularization and soft tissue growth. Cross-linking strategies help maintain mechanical integrity during expansion, while gradual biodegradation minimizes complications like scarring or foreign body response. This approach offers a customizable and biologically integrated alternative to traditional silicone expanders, with potential to improve outcomes in reconstructive surgery. Further research is needed to optimize bioink formulations and validate long-term in vivo performance.

Biodegradable hydrogels are emerging as promising materials in tissue engineering, especially for minimally invasive, load-bearing tissue reconstruction. A newly developed injectable hydrogel made by photo-crosslinking two biomacromolecules - PLGA-APEG and methacrylated gellan gum (GG-MA) - exhibits high mechanical strength, including a compressive stress of 0.53 MPa and strong fracture resistance. When loaded with adipose-derived stem cells (ASCs) and injected subcutaneously in mice, the hydrogel successfully gelled in place with good cell viability (up to 84%) and biocompatibility, without causing inflammation. The hydrogel also showed controlled biodegradation, degrading about 60% over 11 weeks while supporting tissue infiltration. These properties make such injectable, biodegradable hydrogels highly suitable candidates for next-generation STEs that require strength, biocompatibility, and ease of use [137].

## Conclusion

The ideal approach for soft tissue expansion in regenerative dentistry should be effective, well-tolerated by patients, and cost-efficient. Hydrogels used in STEs must maximize effective tissue expansion while minimizing toxicity. While most current research on STEs has focused on subcutaneous implantation, this has limited the understanding of their effects on bone tissue. A key challenge remains in designing a patient-compliant and economic method for soft tissue expansion prior to bone augmentation. Clinically, understanding the pressure thresholds, insertion planes, and material properties of STEs is crucial for optimizing outcomes and preventing complications such as bone resorption, perforation, and inflammation. Appropriate selection of expansion pressure and placement (submucosal vs. subperiosteal) can help clinicians tailor treatment to the patient’s anatomical and reconstructive needs. If the characteristics, advantages, and disadvantages of STEs are well understood and appropriately applied, they can lead to favorable outcomes in reconstructive surgery. Furthermore, the development of minimally invasive, injectable, and biodegradable hydrogel-based expanders may significantly improve clinical workflow and patient comfort, providing clinicians with more predictable and versatile tools for pre-augmentation soft tissue management.

## Data Availability

No datasets were generated or analysed during the current study.

## Abbreviations

STE: Soft tissue expander
ECM: Etracellular matrix
FACs: Focal adhesion complexes
ABH: Alveolar bone height
EGF: Epidermal growth factor
TGF: Transforming growth factor
FGF: Fibroblast growth factor
VEGF: Vascular endothelial growth factor
PDGF: Platelet-derived growth factor
CTGF: Connective tissue growth factor
IL: Interleukin
TNF: Tumor necrosis factor
cAMP: cyclic adenosine monophosphate
IP: Inositol phosphate
PL: Phospholipid
VP: Vinyl pyrrolidone
MMA: Methacrylate
PEGDA: Poly (ethylene glycol)-diacrylate
HEMA: Hydroxyethyl methacrylate
PLGA: Poly (lactide-co-glycolide)
TET: Tunneling graft
TEG: Traditional bone graft
